# Deformation behavior of dragonfly-inspired nodus structured wing in gliding flight through experimental visualization approach

**DOI:** 10.1038/s41598-018-24237-x

**Published:** 2018-04-10

**Authors:** Sheng Zhang, Yuta Sunami, Hiromu Hashimoto

**Affiliations:** 1 Micro/Nano Technology Center, Tokai University, 4-1-1 Kitakaname, Hiratsuka-city, Kanagawa 259-1292 Japan; 2 Department of Mechanical Engineering, Tokai University, 4-1-1 Kitakaname, Hiratsuka-city, Kanagawa 259-1292 Japan

## Abstract

Dragonfly has excellent flight performance and maneuverability due to the complex vein structure of wing. In this research, nodus as an important structural element of the dragonfly wing is investigated through an experimental visualization approach. Three vein structures were fabricated as, open-nodus structure, closed-nodus structure (with a flex-limiter) and rigid wing. The samples were conducted in a wind tunnel with a high speed camera to visualize the deformation of wing structure in order to study the function of nodus structured wing in gliding flight. According to the experimental results, nodus has a great influence on the flexibility of the wing structure. Moreover, the closed-nodus wing (with a flex-limiter) enables the vein structure to be flexible without losing the strength and rigidity of the joint. These findings enhance the knowledge of insect-inspired nodus structured wing and facilitate the application of Micro Air Vehicle (MAV) in gliding flight.

## Introduction

Biomimetic is an interesting and prominent area that has great potential in solving many technical problems in science, which have inspired us to developing the special abilities over the original material properties. In the case of aerodynamics performance, insects’ great flight ability has attracted much attentions in many fields, especially the study of micro air vehicles (MAV)^1^. For instance, the investigation of power consumption in hovering flight of fruit flies is carried out by using instantaneous flow visualizations and force measurements^2^. With the verification of instantaneous force measurements consists of laser interferometry, flow visualization is able to predict the forces generated during the flight motion. In addition, the unsteady mechanism of insects in flapping-wing flight is also investigated, and the force generation of the beating wing is explained by classical aerodynamic theory^3,4^.

In a more specific case, the dragonfly wings are extremely light and corrugated to have high stiffness and strength with excellent aerodynamic performance^5^. Due to the geometrical asymmetry of the wing structure, the deformation is smaller during the downstroke compared with the upstroke movement. In addition, the structural natural vibration of dragonfly wing in vacuum is approximately 4.8 times higher than the natural flapping frequency of dragonflies in hovering flight. The micro air vehicles (MAV) may benefit from this finding to design more efficient wings in flapping motion. In addition, the veins are considered as one of the main structure components of dragonfly wings, moreover, some joints are having high elasticity and connected by resilin^6^. In some research, structural dynamic modification is used to create a simplified model through spatial network analysis approach, and the wing pattern is reduced to meet the fabrication tolerance^7^.

Further investigation on the wing structure is conducted on a nodus wing model by Naka and Hashimoto^8^. In the research, the model imitates the nodus structure of the dragonfly wing, and using fluid-structure interaction analysis to study the effect of passive wing deformation on aerodynamic force. Due to the numerical analysis, the wing is passively deformed on the tip side from the nodus and generates more aerodynamic force in the flapping flight. Flexible wing is more suitable for flapping, but not for gliding flight, the dragonfly wings possess an appropriate level of flexibility and stiffness to have an excellent aerodynamic performance in both case of flapping and gliding flights. This phenomenon is benefited by the deformation of the nodus structured wing.

Most studies have been conducted in FEM (Finite Element Method) to simulate the deformation of the nodus structured wing^9–11^. However, the role of nodus in gliding flight has not been investigated through an experimental approach, especially in visualized tests. In this research, 3D printed biomimetic dragonfly wing structures are fabricated as, open-nodus, closed-nodus and rigid wing. In order to study the function of nodus structure in gliding flight, the samples were conducted in a wind tunnel with high-speed camera to visualize the deformation of wing.

### Structure analysis of a dragonfly wing

**Figure 1:**
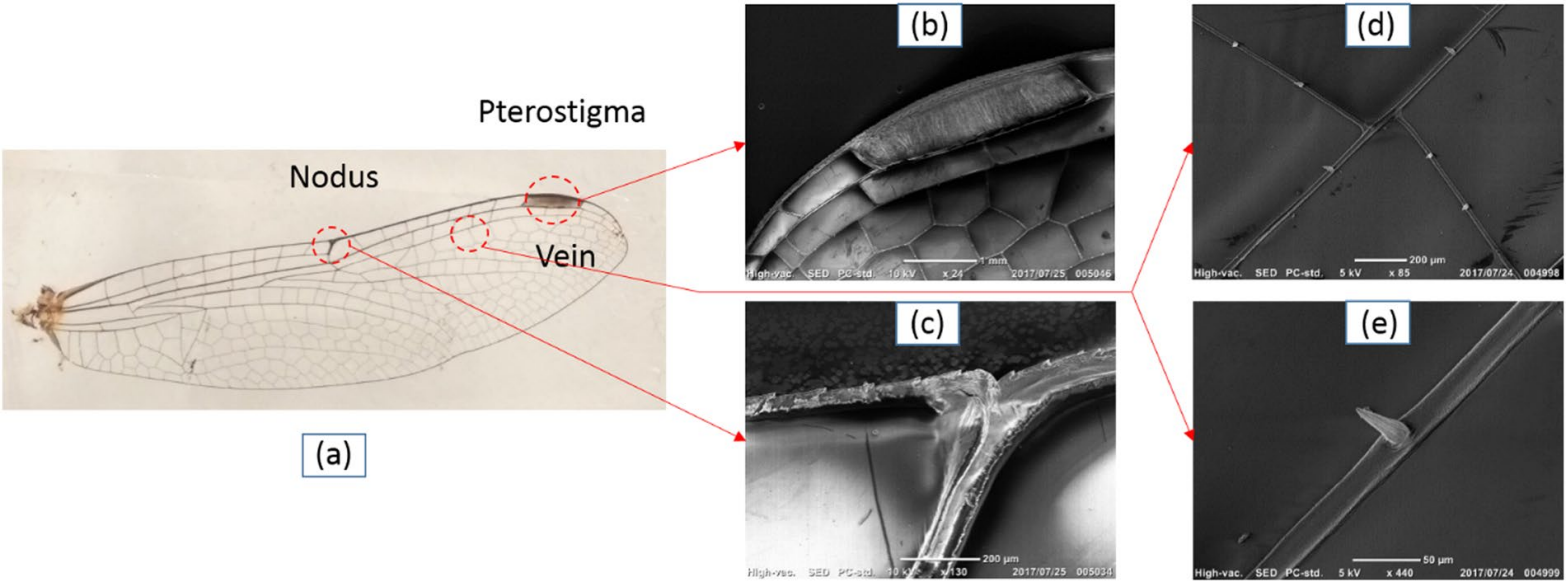
Photograph of a typical dragonfly in Libellulidae family (**a**) with SEM images of detailed wing structure (**b**) pterostigma, (**c**) nodus, (**d**) vein and cells, (**e**) spike on the vein.

The dragonfly studied in this research is a typical dragonfly that belongs to Libellulidae family. The detailed forewing structure is shown in Fig. 1. The length and width of the forewing is 35 mm and 9 mm, respectively. The wing consists of two main parts: vein and membrane. These longitudinal veins create a basic framework of the wing structure that provides two major functions: (1) support for the membrane; (2) bear the bending and twisting moments applied to the wing^12^. The function of membrane is to act as a physical barrier to the passage of air. According to the research conducted by Wooton, the functional morphology of the wings (corrugated wings) of odonatan is reviewed in details^13,14^. Dragonfly wings show complete corrugation with the stems of the main longitudinal veins, and the composite veins are linked at the nodus. In addition, reinforcement can be provided by the cross-veins of the nodus, and its major function is shock-absorption.

The network of veins forms a group of small cells, and these veins stiffen and strengthen the wing structure against bending and twisting moments (refer to Fig. 1d). According to the SEM images of the wing, some unique structural patterns are observed, for example, thickened cells (named pterostigam) near the wing tip are heavier and larger than the other cells (refer to Fig. 1b). Rows of pillar-like spikes are observed on the surface of veins (refer to Fig. 1e). More importantly, a node is observed near the middle of the vein structure (refer to Fig. 1c). Many Finite Element Method (FEM) studies have been done to study the role of the nodus structure in aerodynamic performance, however, rare of them conduct the study through experimental approach^10,11^.

### Sample preparation

**Figure 2:**
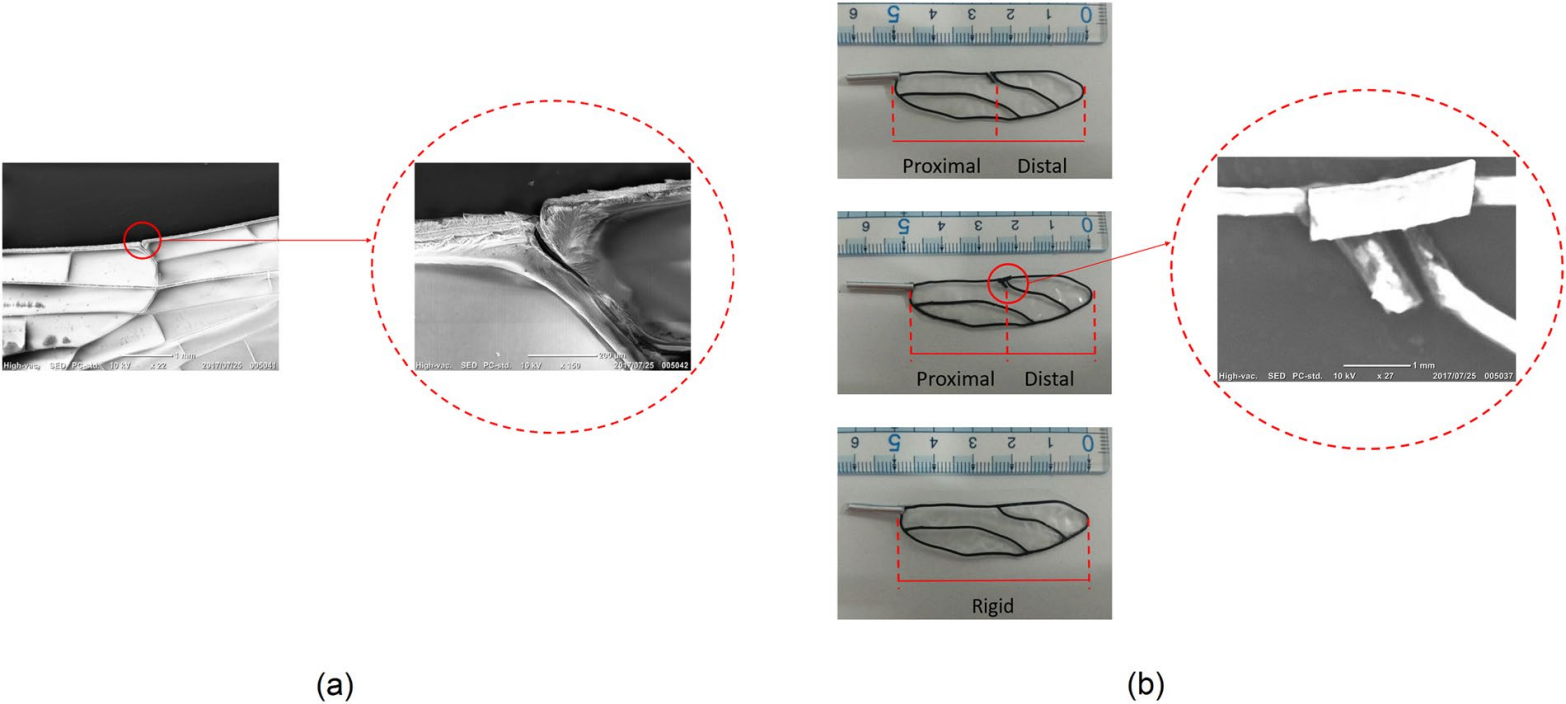
(**a**) SEM images of nodus of dragonfly wing; and (**b**) 3D printed dragonfly wing structure: open-nodus (top-left), closed-nodus with a flex-limiter (middle-left) and rigid wing (bottom-left).

In this research, the objective is to investigate the function of nodus through an experimental approach. To do so, three dragonfly-mimic vein structured wings were designed and fabricated by a commercial 3D printer (Anycubic I3 MEGA) as: open-nodus, closed-nodus and rigid wing (refer to Fig. 2b). Due to the limitations of 3D printing technology, the structure of the wing nodus is simplified compared to the structures of the actual nodus of dragonfly. The vein material is a bio-friendly thermoplastic aliphatic polyester material, polyactide (PLA). In addition, each vein structure is filmed with a polyvinyl alcohol (PVA) material to mimic the membrane of the dragonfly wing. According to the SEM image of the dragonfly wing (forewing), the structure has a distinct node (nodus) that divides the wing into two parts: proximal (pre-nodal) and distal (post-nodal) part (refer to Fig. 2a). In general, the dragonfly wings have nodus that span between 47% and 60% of the wingspan^11^. In this research, the nodus is located 55% of the wingspan for both nodus structured wing (open-nodus and closed-nodus). For the closed-nodus wing, the vein structure is modified based on the main structure of the open-nodus wing, in addition, a flex-limiter is added to the nodus. This unique flex-limiter is fixed on the proximal part and free of distal part to have a controlled flexibility to prevent excessive wing bending (refer to Fig. 2b).

### Wind tunnel set-up

**Figure 3:**
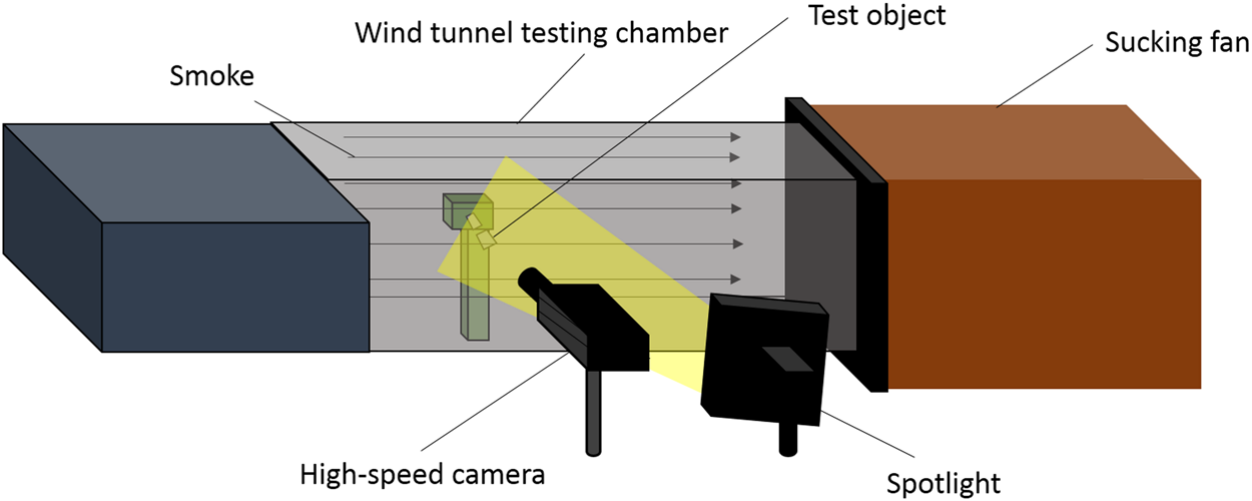
Schematic diagram of wind tunnel test.

The visualization of the gliding flight on the fabricated wing structures were conducted in a wind tunnel chamber (refer to Fig. 3). During the test, the wind speed gradually increased from low to high, and the wind speed was controlled by a fan inverter (fan strength ranged from 0 to 100). The angle of attack for each wing sample is 0°. The wind speed was measured by an anemometer during the experiments. Six wind speeds were performed for each wing sample: 3.4 m/s ± 0.3 (Reynolds number = 3500), 4.6 ± 0.3 m/s (Reynolds number = 5000), 5.9 ± 0.4 m/s (Reynolds number = 6000), 7.1 ± 0.3 m/s (Reynolds number = 7500), 8.4 m/s ± 0.5 (Reynolds number = 9000), 9.5 m/s ± 0.5 (Reynolds number = 10000), and categorized into three regimes as low (3.4 m/s and 4.6 m/s), medium (5.9 m/s and 7.1 m/s) and high (8.4 m/s and 9.5 m/s). The ambient testing temperature is 24.3 °C. In the test chamber, the wing sample was fixed on the stage, and a high-speed camera with smoke machine was used during the experiments to observe the deformation of the wing structure.

## Results and Discussion

The purpose of this research is to investigate the deformation behavior of nodus on vein structured wing through an experimental visualization approach. During the wind tunnel tests, the wing structure deformation of all samples are unnoticeable by visual observation at the low wind speed regime (3.4 m/s and 4.6 m/s), except for the open-nodus wing sample with slight vibration at wind speed of 4.6 m/s. However, once the medium regime of wind speed reached at 5.9 m/s, the wing structure began to vibrate slightly for the rigid wing. At the wind speed of 7.1 m/s, the vibration on the wing tip started to get intense and tended to flap for the rigid wing sample. For the open-nodus wing sample with the same wind speed (7.1 m/s), the vibration became intense and flap motion was detected. However, for the closed-nodus wing sample, only slight vibration was observed at the wing tip.

**Figure 4:**
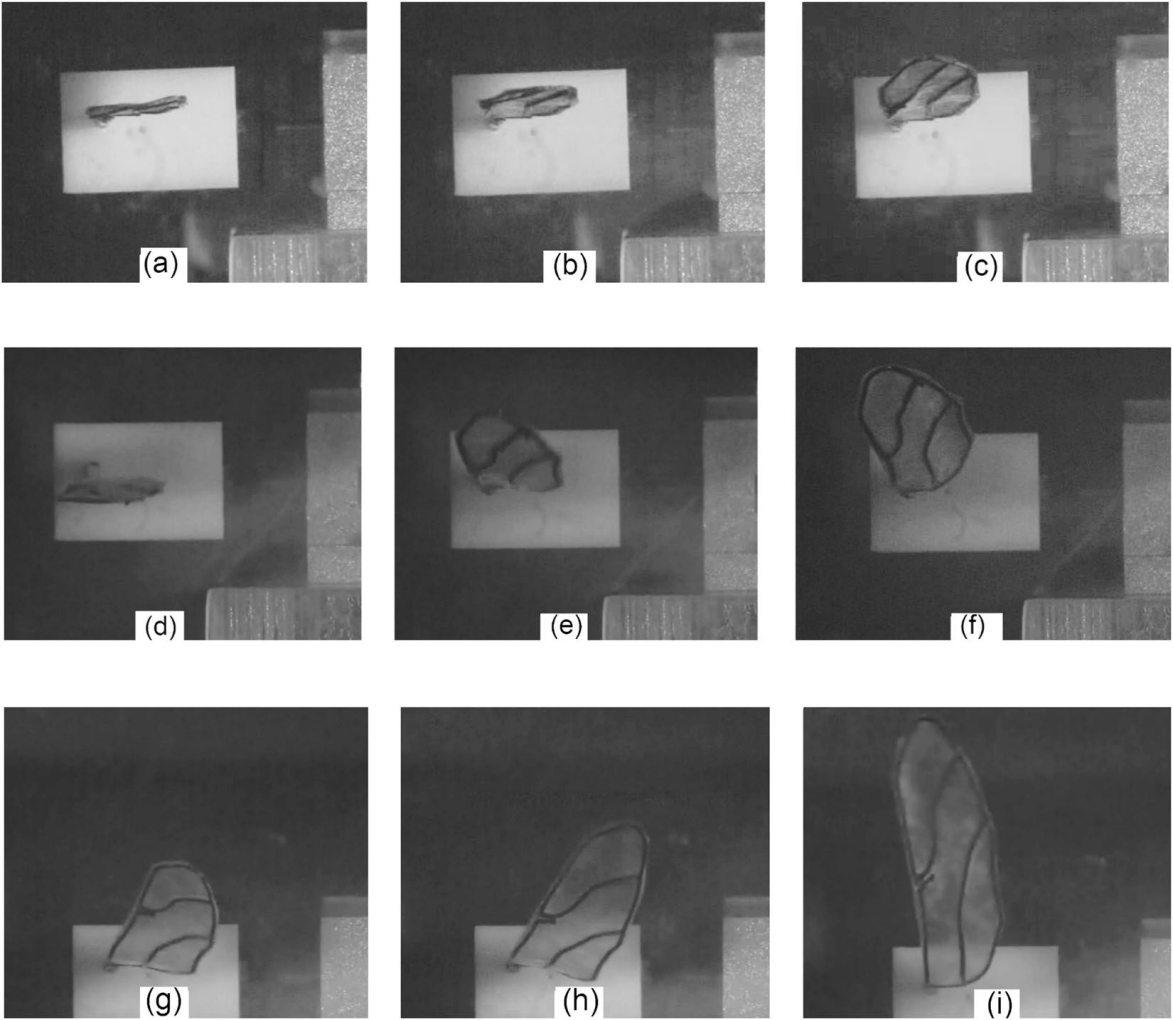
Images obtained by high-speed camera at wind speed 9.5 m/s, (**a**) initial deformation of closed-nodus wing; (**b**) deformation transition of closed-nodus wing; (**c**) maximum deformation of closed-nodus wing; (**d**) initial deformation of rigid wing; (**e**) deformation transition of rigid wing; (**f**) maximum deformation of rigid wing; (**g**) initial deformation of open-nodus wing; (**h**) deformation transition of open-nodus wing; (**i**) maximum deformation of open-nodus wing.

**Figure 5:**
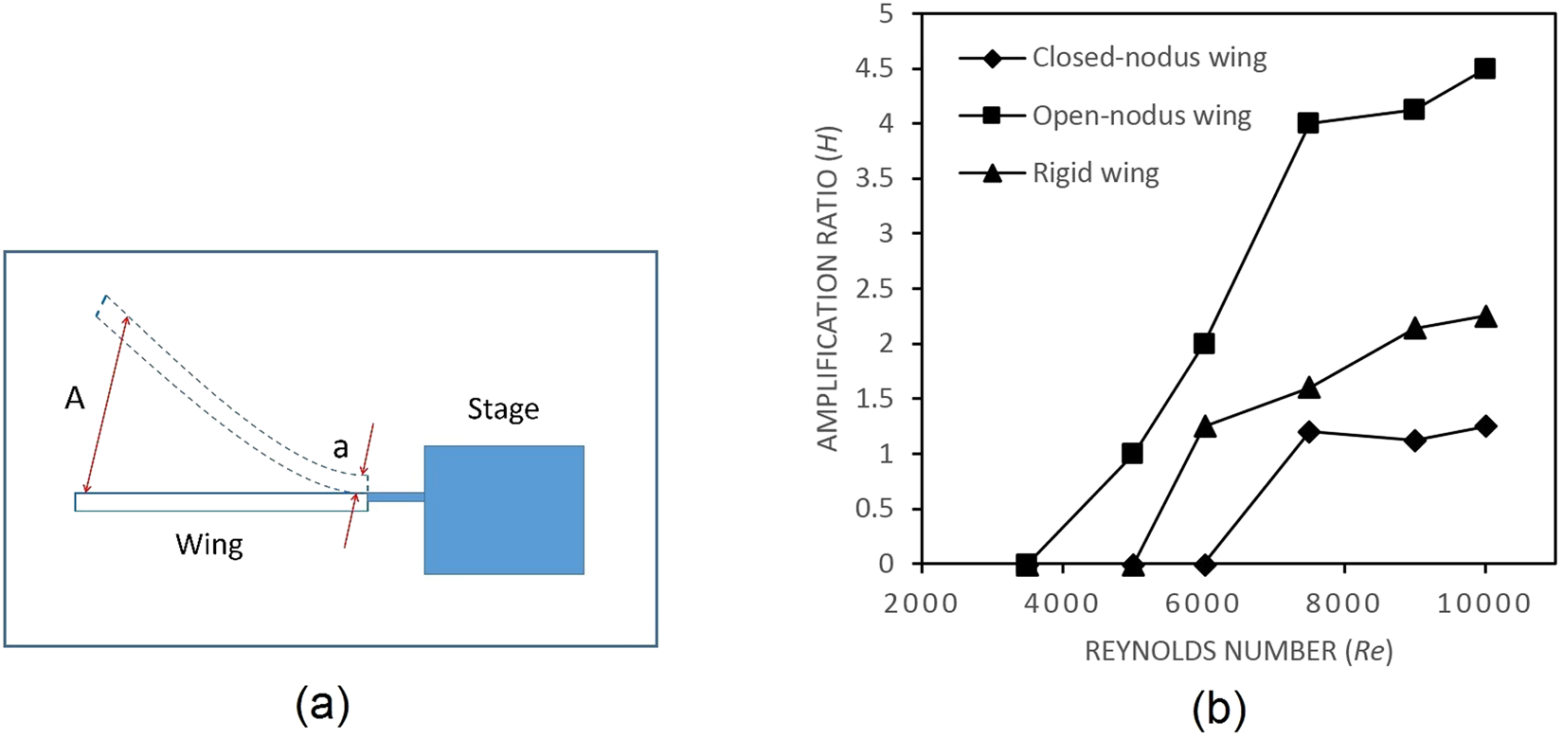
(**a**) Schematic diagram of amplification ratio *H*; (**b**) calculated amplification ratio *H* of all samples at Reynolds number *Re* ranged from 3500 to 10000.

In the high wind speed regimen (8.4 m/s and 9.5 m/s), all wing samples were structurally deformed and intense flapping motions were observed (refer to Fig. 4). The wing deformation process can be recognized as the initial deformation (start to deform), the deformation transition (transition to maximum deformation) and the maximum deformation position. The highest bending positions of all wing samples were observed at the wing speed of 9.5 m/s, and the amplification ratio *H* can be determined from two displacements according to the following equation^15^:

1 $$H=\frac{A}{a}$$

where *A* and *a* are the displacements of the wing tip and wing base, respectively (refer to Fig. 5).

Based on the images captured by a high-speed camera (5000 frames per second), as shown in Fig. 5, the amplification ratio *H* on each wing structure can be visualized. The calculated amplification ratio *H* is shown in Fig. 5(b), for instance, the values of *H* for closed-nodus wing, open-nodus wing and rigid wing at wind speed of 9.5 m/s (Reynolds number = 10000) are 1.25, 4.5 and 2.25, respectively. The open-nodus wing sample has the highest amplification ratio (*H = 4.5*), which is experiencing an excessive over-bending at the wind speed of 9.5 m/s. Compare to the open-nodus structure, the rigid wing sample shows less flexibility (smaller wing deformation) and lower amplification ratio (*H* = 2.25).

The open-nodus structure is observed to have the highest flexibility among all samples. According to the research, the nodus on the dragonfly wing separates the structure intro two parts, proximal and distal, as the result, the flexibility of the wing is increased and more suitable for flapping flight^16^. In this study, the open-nodus structured wing has the same function to separate the wing into pre-nodal and post-nodal sections, and providing much greater flexibility. However, the open-nodus is completely divided into two isolated half-nodes, which made the wing structure easy to deform and over-bend. Moreover, this high-flexibility can cause high amplification ratio which is not suitable for gliding flight.

**Figure 6:**
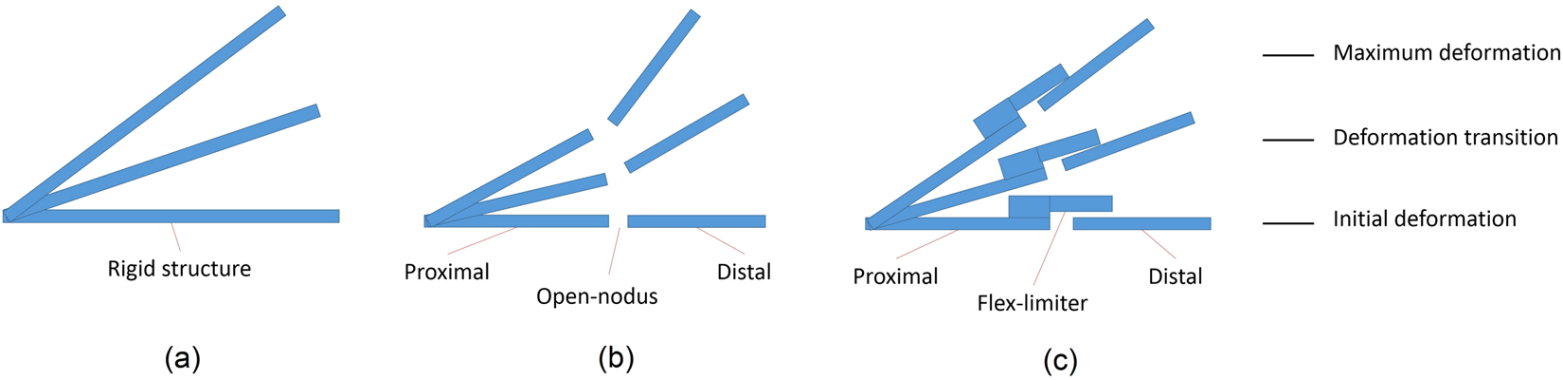
Schematic illustration of deformation process of (**a**) rigid wing, (**b**) open-nodus structured wing and (**c**) closed-nodus structured wing.

For the closed-nodus structured wing, a flex-limiter is designed to attach on the nodus to eliminate the over-bending problem at high wind speed (refer to Fig. 6). Based on the wind tunnel tests, the closed-nodus structure has the least deformation and the lowest amplification ratio among all three samples in this research. The flex-limiter restrains the flexibility of the wing structure by limiting the upward bending on the distal (post-nodal) part of the wing. One end of the flex-limiter is fixed on the proximal part of the wing, and other end is freely attached (not fixed) on the distal part of the wing. Therefore, the flexibility of both proximal and distal parts of the wing are not eliminated, however, the bending angle between these two parts is limited to prevent excessive bending deformation (refer to Fig. 6c). This finding is consistent with the work conducted by Wootton, the nodus is acting as a shock-absorber to relieve potentially destructive energy of the twisting wing^13^. In the contrary, the distal part of open-nodus structured wing has less deformation restrain of proximal part, as the result, the entire structure is highly flexible and easy to have over-bending deformation in gliding flight.

**Figure 7:**
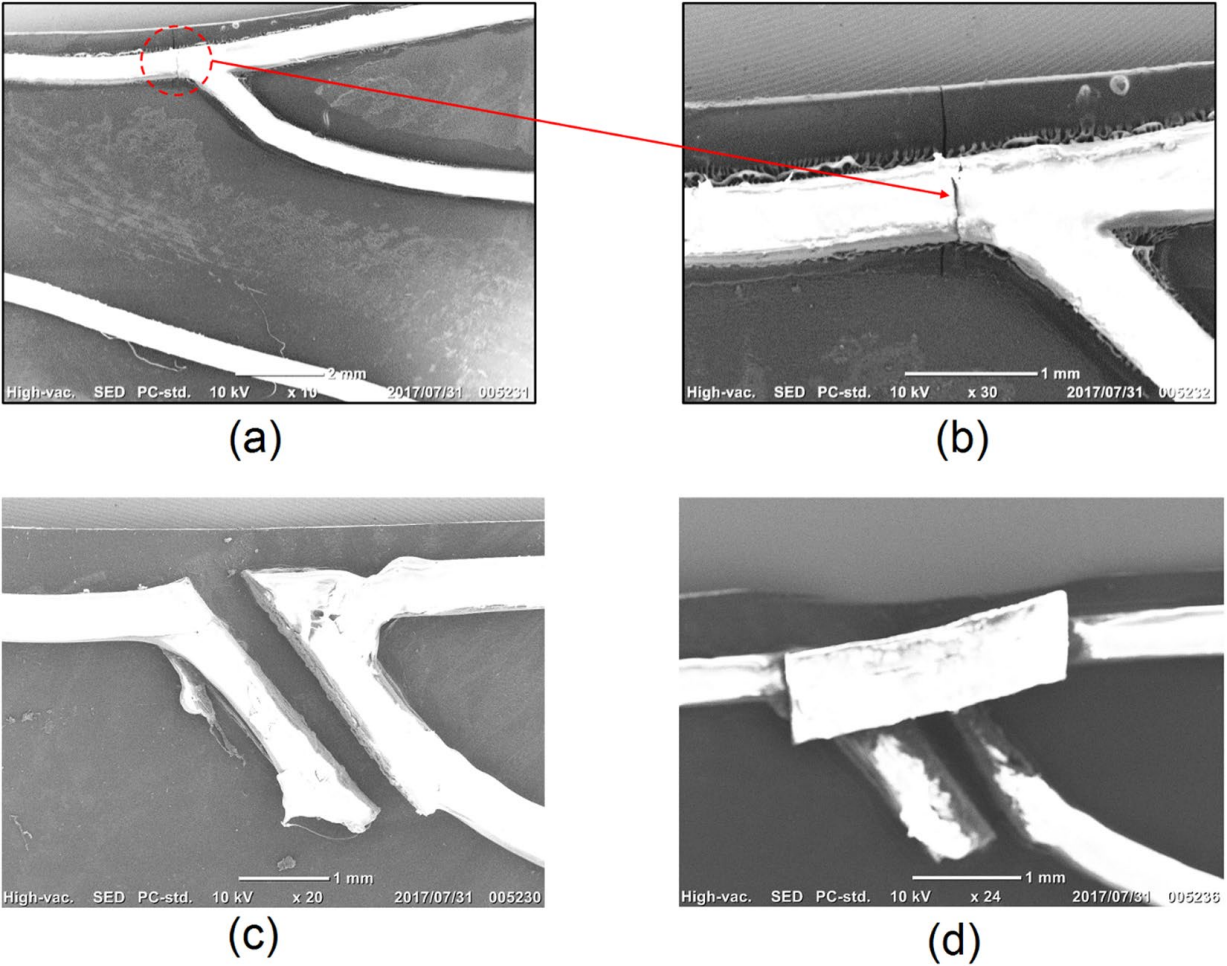
SEM images of wing samples after wind tunnel tests, (**a**) fatigue crack initiation on rigid wing, (**b**) zoomed image of crack on rigid wing; (**c**) no crack observed on same location for open-nodus wing; (**d**) no crack observed on the same locatio for closed-nodus wing.

After the wind tunnel experiments, all wing samples are examined by SEM carefully. A fatigue crack initiation has been observed on the vein and membrane of rigid wing sample (refer to Fig. 7a,b). The crack is located approximately at the center of the structure subjected to the most bending and twisting moments. For the open-nodus and closed-nodus structure, no sign of crack initiation is observed at the same location of rigid wing or other locations on the vein structure. In order to validate this finding, an additional experiment was conducted on nine wing samples, three for each wing structure. All samples were tested in the wind tunnel chamber at the wind speed of 9.5 m/s ± 0.5 for one hour. After the tests, the fatigue cracks located at the center of the wing structure are observed on two rigid wing samples, however, no sign of crack is observed on the wing samples with nodus. This additional test further confirm that the nodus is able to prevent the occurrence of fatigue crack. In addition, the nodus serves as a shock-absorber to cope with the local stresses between the two sections of the leading edge spar with different mechanical properties. This is consistent with the FEM simulation (ABAQUS) conducted by Rajabi *et al*., the orientation of the leading edges changes at the nodus, and the areas around nodus bear the highest bending and twisting moments^11^. In a more specific FEM analysis conducted by Fauziyah *et al*., the mechanical characterization of wing nodus from the Libellulidae family of dragonfly is investigated, and the simulation results show that the strain energy highly localized in the nodus compared to other regions of the wing. The nodus is able to protect the wing structure from fracture during flight^17^. These FEM results in the literature are consistent with our experimental results in this study.

In conclusion, the nodus strongly affects the deformation of a typical vein structure. The deformation effect and flexibility of the vein structure can be controlled by adding a flex-limiter. Moreover, the closed-nodus structured wing has the least amplification ratio with controlled flexibility, which is the most suitable structure for gliding flight. The open-nodus structured wing has the highest amplification ratio, and this high flexibility may be applied to flapping-only flight. The reference sample, rigid wing, shows medium amplification ratio and wing deformation among samples. Furthermore, the vein structure without nodus is relatively fragile compared to the vein structure with nodus.

## Conclusion

In this research, the vein structures of dragonfly wings were fabricated as open-nodus, closed-nodus and rigid wing with same material (PLA) and membrane (PVA). Based on the wind tunnel tests in low, medium and high wind speeds, the open-nodus structured wing has the highest flexibility based on the visulization approach with a calculated amplification ratio of 4.5 at wind speed of 9.5 m/s. However, by adding a flex-limiter to the same vein structure, the flexibility is reduced with a calculated amplification ratio of 1.25. The rigid wing (vein structure only), as a reference sample, has the medium flexibility with a calculated amplification ratio of 2.25. The overall results are summarized as follows:

- The nodus strongly affects the deformation of a typical vein structure.
- Flex-limiter is able to control the nodus effect on flexibility of vein structured wing.
- Closed-nodus (with flex-limiter) structured wing is suitable for gliding flight due to its low amplification ratio.
- Nodus prevnets fracture on the vein structure during flight.

Overall, the closed-nodus wing (with a flex-limiter) enables the vein structure to be flexible without losing the strength and rigidity of the joint. These findings enhance the knowledge of insect-inspired nodus structured wing and facilitate the application of Micro Air Vehicle (MAV) in gliding flight. For the future study, a more realistic nodus with different fabrication methods will be investigated to further understand the structure and function of the corrugated wing.
